# Exploring the End-Use Quality Potential of a Collection of Spanish Bread Wheat Landraces

**DOI:** 10.3390/plants10040620

**Published:** 2021-03-24

**Authors:** Matilde López-Fernández, Laura Pascual, Isabel Faci, Mario Fernández, Magdalena Ruiz, Elena Benavente, Patricia Giraldo

**Affiliations:** 1 Department of Biotechnology-Plant Biology, School of Agricultural, Food and Biosystems Engineering, Universidad Politécnica de Madrid, 28040 Madrid, Spain; matilde.lopez@upm.es (M.L.-F.); laura.pascual@upm.es (L.P.); i.faci@alumnos.upm.es (I.F.); mario.fernandez.gonzalez@upm.es (M.F.); e.benavente@upm.es (E.B.); 2 National Plant Genetic Resources Centre, National Institute for Agricultural and Food Research and Technology, 28800 Alcalá de Henares, Spain; mruiz@inia.es

**Keywords:** *Triticum aestivum*, wheat landraces, genetic diversity, HMW glutenins, puroindolines, gluten quality

## Abstract

Modern plant-breeding practices have narrowed the genetic base of wheat, such that there is a need to introduce new germplasms with underexploited diversity into breeding programs. Wheat landraces are a very valuable resource when searching for genetic variation, which not only possess increased adaptability, but also quality-related traits. Several studies have shown a wide genetic diversity in Spanish wheat landraces compared to other germplasm collections; therefore, the main objective of this study is to analyze the variability in a collection of 189 landraces from the Spanish National Plant Genetic Resources Centre (Centro de Recursos Fitogenéticos, CRF-INIA, Alcalá de Henares), in relation to end-use quality traits. We characterized the whole collection for high-molecular-weight glutenin and puroindoline allelic composition, and for gluten strength. In addition, grain protein content, grains per spike, and thousand kernel weight were evaluated in samples from four-year field trials. The relationship between glutenin composition and quality was evaluated, and some alleles strongly associated with high quality were identified in the collection, some of them specific for Iberian landraces. The results also show the presence of novel variability within high-molecular-weight glutenin and puroindolines, which needs to be characterized further in order to assess its influence on wheat quality. In addition, a set of landraces showing outstanding values for gluten quality and a good agronomic performance was selected for testing in field trials in order to evaluate the suitability of their direct use in cropping systems.

## 1. Introduction

Wheat is one of the three main crops grown across the world. It covers 214 million hectares and represents a quarter of total cereal production, with 765 million tons produced in 2019 [1]. Natural hybridization events between different species have led to the wheat that is currently cultivated [2], including the hexaploid species *Triticum aestivum* spp. *vulgare* (Vill.) (bread wheat, 2n = 6x = 42, AABBDD), which represents roughly 90% to 95% of the total production. Bread wheat is an essential crop for humans; it provides 20% of total protein and calories in daily intake through a variety of geographically derived products (leavened breads, flatbreads, noodles, cookies, etc.) [3]. Currently, wheat production must meet the increasing needs of a growing human population while taking into account the uncertain climate conditions and the demand for more sustainable agriculture. Since the Green Revolution in the 1960s, the genetic base of wheat has been narrowed because only a small set of elite cultivars has been used for breeding [4]. In this context, wheat landraces, specifically adapted to their region of origin and traditionally grown with less inputs, represent an important source of genetic variability [5,6,7]. Several studies have yet to successfully identify interesting landraces that could widen the gene pools of modern cultivars by adding underexploited diversity to wheat breeding programs [8,9,10,11].

Although the main current breeding goal is the development of stable yield cultivars, most wheat production is used for human consumption and, thus, wheat end-use quality is always a key breeding target [12]. The main determinants of wheat quality are endosperm proteins, in terms of quantity and quality. Grain Protein Content (GPC) is a complex trait affecting both nutritional value and dough rheological properties and is highly dependent on environmental conditions [13,14,15]. Besides protein content, the main factor affecting end-use quality is the prolamin composition of the wheat grain, especially the High Molecular Weight glutenin (HMW-Gs) fraction [16]. HMW-Gs are encoded by the complex *Glu-1* loci, located close to the centromere on the long arm of homoeologous group 1 chromosomes (*Glu-A1*, *Glu-B1*, and *Glu-D1*) [17]. These loci include two closely linked genes that encode for two polypeptides: the x-type glutenin subunit and the y-type glutenin subunit [16,18]. In general, the *Glu-A1* locus encodes one or no subunit, the *Glu-B1* locus encodes one or two subunits, and the *Glu-D1* locus encodes two subunits [19]; therefore, bread wheat cultivars present three to five different HMW-Gs subunits. The relationship between HMW-Gs composition and gluten strength was established many years ago [16,20,21], and several studies have ranked HMW-Gs alleles according to their influence on flour quality [16,22]. The *Glu-D1d*, *Glu-B1b*, and *Glu-B1c* alleles, strongly associated with high end-use quality, have been described as the most suitable to breeds for end-use value [23]. However, the identification of novel glutenin alleles and allelic combinations with eventual superior quality will help widen the wheat breeding gene pool and is thus an important breeding target. 

Besides endosperm proteins, endosperm texture (or grain hardness) is also an important quality factor in bread wheat. Kernel texture, directly related to the amount of damaged starch produced during the milling process, is used to classify wheats as either hard or soft. Hard wheat grains produce more damaged starch, which results in higher water absorption when doughs are formed [24,25] (more suitable for leavened breads), while soft wheat kernels are easily fractured, resulting in fine flour with less damaged starch (preferred for cookies, cakes, and pastries) [26]. Grain hardness is controlled by two genes at the *hardness* locus (*Ha*), located on the short arm of chromosome 5D: the *Pina-D1* gene and the *Pinb-D1* gene, which code for the puroindoline a and puroindoline b proteins, respectively [25,27]. The wild-type form of these genes results in soft-textured grains, while the absence of mutation(s) in either or both genes results in hard wheats [28]. As the major causal genes of kernel hardness, puroindolines with allelic diversities have been extensively investigated in a wide range of wheat germplasm collections, and more than 50 alleles have been described so far [29,30,31,32,33].

Landraces might harbor new end-use quality variability for breeding programs, but their proper use requires deep characterization not only for quality, but also for agronomic traits [34]. However, the main burden when breeding for quality is the fact that some key quality-related traits, such as grain protein content, have been negatively correlated with yield [35]. Yield is a complex trait determined by multiple quantitative loci that interact with each other and with the environment [36]. This trait can be dissected into different components, including Thousand Grain Weight (TKW) and Grain Number Per Spike (GN) [37]. TKW is not only an essential yield component but also an important quality trait that interacts with other quality-related traits, such as protein content, to which it is negatively correlated [35]. GN has been shown to be less sensitive to environmental changes than other yield components, especially in landraces [38]. 

In Spain, the widest collection of wheat landraces is composed of 522 Spanish accessions of *Triticum aestivum* ssp. *vulgare* (Vill.), conserved in the Spanish National Plant Genetic Resources Centre, Centro de Recursos Fitogenéticos, INIA, Alcalá de Henares (CRF-INIA). Given the high agroclimatic diversity of the Iberian Peninsula, these landraces, which are locally adapted, represent a large source of genetic variability [39,40,41]. Moreover, in relation to quality, several new HMW-Gs alleles have been detected in Spanish landraces [42,43]. A recent study explored the genetic structure of a subset of this collection and determined the existence of four different populations, representing different gene pools for plant breeding [41]. According to the pedigrees of modern wheat cultivars, these landraces have not played a significant role in wheat improvement [44]. Thus, the aim of the current study is to evaluate the genotypic and phenotypic diversities in relation to the quality and yield components of an assortment of 189 Spanish bread wheat landraces and old cultivars compared to 18 modern bread wheat varieties. Evaluation of this material in field trials may provide valuable information about its potential for wheat quality breeding.

## 2. Results

The germplasm bank codes of the studied landraces along with the commercial varieties’ names are given in Supplementary Table S1, which also includes their genetic profile and the data from the field trials.

### 2.1. Genotypic Characterization of Bread Wheat Landraces

In order to facilitate the exploitation of Spanish wheat landraces in breeding programs, we determined their allelic profile for quality-related genes: puroindolines and HMW-Gs subunits.

#### 2.1.1. Puroindoline Genotyping

The allelic variation in puroindoline genes was determined in the full set by comparing the gene sequences to alleles available in the GenBank database. Most landraces (95.70%) carried the wild-type soft *a* allele of the *Pina-D1* gene, with the *Pina-D1b* hard-type allele only being present in eight accessions (Table 1). For the *Pina-D1a* landraces, the most frequent *Pinb-D1* allele was the wild-type (soft-type) allele *Pinb-D1a* (80.65%). The frequencies of the *Pinb-D1b* and *Pinb-D1d* hard-type alleles were low (2.69%). Two additional rare *Pinb-D1* alleles were found. Fifteen landraces carried an allele previously described only in Spanish landraces and spelt wheat, not included in the Catalogue of Gene Symbols, but tentatively classified as the allele *Pinb-D1ad* [45]. This allele has a C/T change in position 271. It leads to an early stop codon in the predicted mature protein (Q91*), which is associated with a hard texture [45]. Three landraces carried a *Pinb-D1* allele that was not previously described. This allele has a G/A change in position 55, which produces an A19T change in the predicted protein sequence. The puroindoline b protein sequence alignment can be found in Figure S1. In the modern varieties (from now on named the reference set), most of the varieties had the *Pina-D1a*/*Pinb-D1a* combination, with the *Pina-D1b*, *Pinb-D1b*, and *Pinb-D1d* alleles being present in the same proportions.

#### 2.1.2. HMW-Gs Subunits Characterization

**Figure 1:**
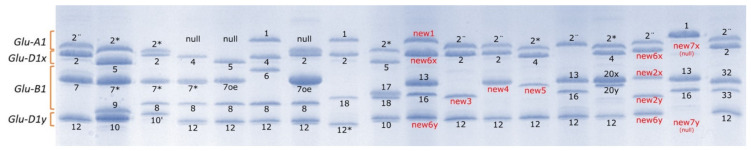
Sodium dodecyl sulfate–polyacrylamide gel electrophoresis (SDS–PAGE) patterns of bread wheat landraces illustrating the variability in high-molecular-weight glutenin subunits (HMW-Gs) observed in the collection under study. The subunits not previously described are highlighted in red.

The total set of varieties was genotyped by sodium dodecyl sulfate–polyacrylamide gel electrophoresis (SDS–PAGE) and PCR for the complex *Glu-1* homoeoloci (see Table S1). The landraces showed a huge degree of variability in HMW-Gs subunit composition, as shown in Figure 1. 

Five different alleles at the *Glu-A1* locus, fourteen different alleles at the *Glu-B1* locus, and nine different alleles at the *Glu-D1* locus were identified in the 189 Spanish landraces analyzed (Table 2). Several new subunits, not corresponding to any allele previously described in the catalogue [46], were also identified: one at the *Glu-A1* locus, four at the *Glu-B1* locus, and two at the *Glu-D1* locus (Table 2 and Figure 1). 

At the *Glu-A1* locus, the most frequent HMW-Gs subunit was the 2* subunit (*b* allele) found in 53.44% of the landraces, followed by subunits 2¨, 1, and null (alleles *y*, *a*, and *c*, respectively) that were found in 17.99%, 16.40%, and 11.64% of the landraces, respectively. The *Glu-B1* locus was the most polymorphic, with nine previously catalogued alleles identified, namely *a*, *d*, *e*, *f*, *aq*, *al*, *am*, *i*, and *u*. Among them, *e*, *f*, and *u* were the most frequent (47.09%, 15.87%, and 13.76%, respectively). Other subunits/alleles were found at a low frequency (from 0.53% for not previously described alleles to 5.82% for the *d* allele). At the *Glu-D1* locus, seven previously described alleles were found, with 2+12 (*a* allele, 67.20%) being the most frequent combination, followed by 4+12 (*c* allele, 23.28%). The *d*, *h*, *j*, *l*, and *u* alleles were rare, being found in less than eight varieties.

In the reference set, most varieties presented the *b* allele at the *Glu-A1* locus; the *b, c*, or *d* alleles at the *Glu-B1* locus; and the *d* allele at the *Glu-D1* locus (Table S1).

### 2.2. Evaluation of End-Use Quality-Related Traits

The landraces and reference set were analyzed for Grain Protein Content (GPC) and gluten strength, measured by the sodium dodecyl sulfate-sedimentation (SDSS) test. These traits were evaluated in samples from four different seasons in landraces and three seasons in the reference set (Table 3). Climatic data from the trial sites were recorded and, according to them, water input was adequate for wheat growing in the 2017–2018 and 2019–2020 seasons (>300 mm [47]). However, 2016–2017 and 2018–2019 were especially dry; thus, the plants likely suffered from drought stress. Moreover, in the 2016–2017 season, the highest temperatures were achieved (Figure S2).

#### 2.2.1. Landrace Performances in Comparison to Reference Varieties 

In landraces, mean GPC was between 11.51% and 17.59%, being significantly higher than in the reference set varieties for the three years when both sets were sown (Table 3). The highest GPC values were recorded in the 2016–2017 and 2018–2019 seasons, the driest ones. With respect to gluten strength, the landraces showed significant lower mean values (37.9 to 58.14 mm) than the reference (52.28 to 87.89 mm) set but a wider range, including some landraces with values higher that the reference set (seasons 2017–2018 and 2019–2020). The lowest mean values were recorded in 2017–2018, although the range variation was comparable between years.

Spearman’s correlation analysis was carried out between traits and between years (see Table S2). As expected, SDSS values showed the highest positive correlation values between years (from 0.65 to 0.85) and GPC showed lower values (from 0.34 to 0.58), as this trait is much more dependent on environmental conditions. No correlation was found between both traits.

#### 2.2.2. Year and Genetic Structure in Relation to GPC and SDSS Values of Landraces

The effect of year and genetic structure ([41], see the Material and Methods section) on the GPC and SDSS values in the set of landraces was evaluated by ANOVA and Kruskal–Wallis tests, respectively.

**Figure 2:**
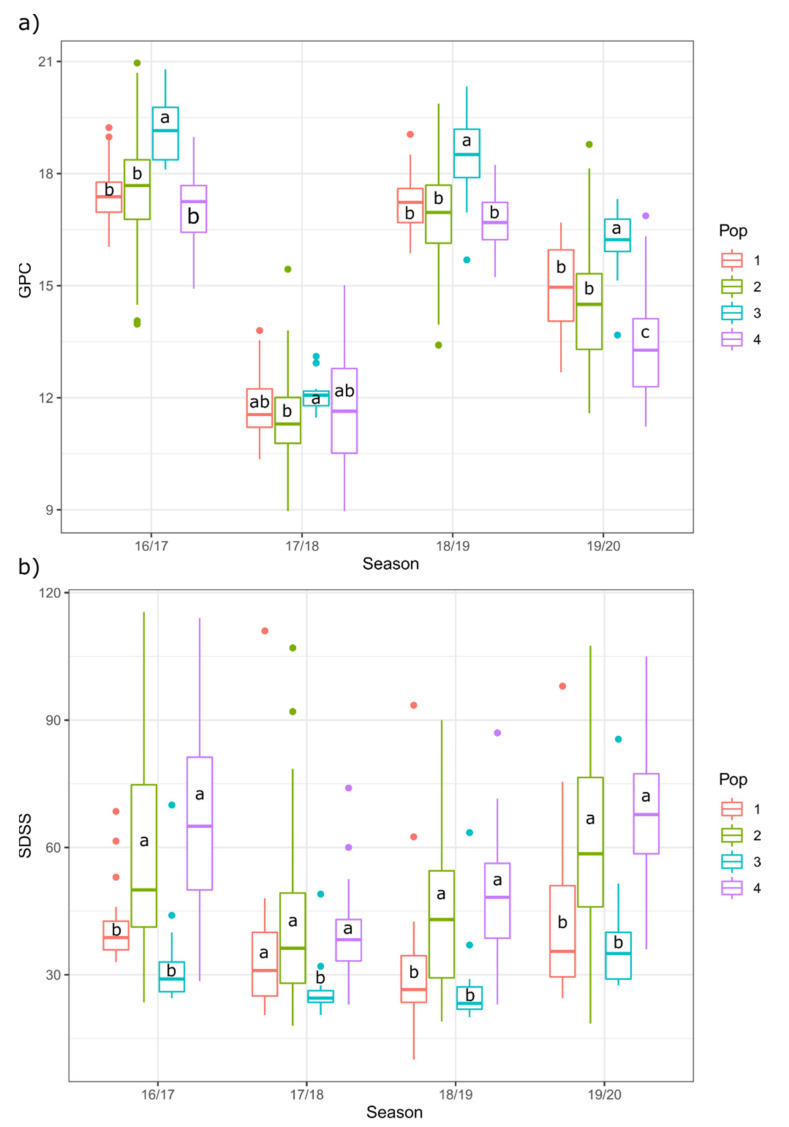
Effect of year and genetic structure of the collection on the (**a**) Grain Protein Content (GPC, %) and (**b**) SDS Sedimentation (SDSS) test values (mm). The results of the Duncan (**a**) or Wilcox (**b**) multiple comparison tests of means are included.

Genetic structure and year had a significant effect on GPC values, and a population × year interaction effect was also observed (Figure 2). The GPC values were higher in dry seasons (2016–2017 and 2018–2019). Genetic population 3 showed significantly higher GPC values than the other populations in three of the seasons; however, in the 2017–2018 season, the rainiest one, this difference was not significant, with the means for GPC in all populations being comparable and low. 

Regarding SDSS values, a genetic structure effect was observed but no population × year effect was detected. Landraces from population 3 showed the lowest values consistently.

### 2.3. HMW-Gs Influence on Gluten Strength

**Figure 3:**
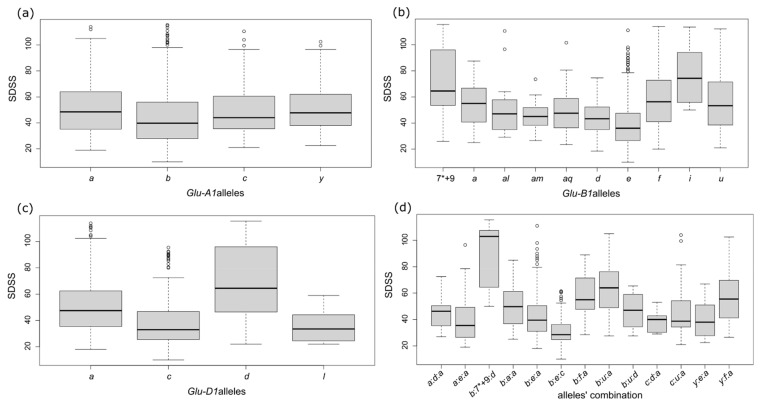
Landrace SDS Sedimentation (SDSS) test values (mm) according to *Glu-1* allele composition: (**a**) *Glu-A1*, (**b**) *Glu-B1*, (**c**) *Glu-D1* and (**d**) allele combination at the three *Glu-1* loci.

A Kruskal–Wallis analysis followed by a Wilcox test were conducted to determine the effect of allelic variation at the *Glu-1* loci on gluten strength measured by the SDSS test. Although landraces with the same HMW-Gs genotype showed a wide range of SDSS values, significant influences were detected for all loci. The highest values corresponded to landraces with *a*, *i*, or *d* alleles at the *Glu-A1*, *Glu-B1*, and *Glu-D1* loci, respectively (Figure 3a–c and Table S3).

At the *Glu-A1* locus, significant differences were found between the *b* and *a*, and *c* and *y* alleles, with the *b* allele being associated with lower mean values. Among the seven alleles analyzed at the *Glu-B1* locus, the *e* allele showed the lowest SDSS values. Significant differences between the *e* allele and the ones with the highest values (*a*, *f*, *I*, *u*, and 7*+9) were found. Regarding this locus, the 7*+9 combination and *i* allele were the most outstanding allelic variants for gluten strength. A significant influence of the *Glu-D1* genotype was also found, with significant differences among the *a*, *c*, *d*, and *j* alleles and with the *d* allele being associated with the highest gluten strength (Figure 3c and Table S4).

The analysis of allelic variation at a single locus facilitates the identification of alleles with a relevant effect on a trait, particularly when the loci under study are highly polymorphic. However, gluten performance is the outcome of the interaction among different alleles. Therefore, after analyzing the influence of each *Glu-1* locus independently, we studied the influence of the allele combinations from the three loci: *Glu-A1*:*Glu-B1*:*Glu-D1* (Figure 3d and Table S5). We analyzed thirteen *Glu-1* combinations presented in at least three accessions. The combination *b*:7*+9:*d* showed the highest SDSS values, which were significantly different from almost any other allele combination. On the contrary, the *a*:*e*:*a, b*:*e*:*a, b*:*e*:*c, c*:*d*:*a*, and *y*:*e*:*a* combinations showed the lowest values (Figure 3d and Table S6).

### 2.4. Evaluation of Yield Components (TKW and GN)

#### 2.4.1. Landrace Performances in Comparison to Modern Varieties 

The landraces and reference set were also characterized for yield components TKW and GN. These traits were recorded in samples from four different seasons in landraces and from three seasons in the reference set (Table 4).

The mean TKW values in landraces were significantly higher than that in the reference set in two of the three years analyzed, attaining the maximum value for both sets in the rainiest season 2017–2018 (Table 4). GN was significantly lower in landraces than in the reference set in the three years analyzed, and the highest values for both sets were achieved in the 2019–2020 season, the mildest one. 

Spearman’s correlation analyses were carried out between traits and between years (see Table S2). GN and TKW showed low correlation values between years (from 0.39 to 0.47 for GN and from 0.35 to 0.57 for TKW) as these traits are very dependent on environmental conditions. 

When the correlation between traits were analyzed, a strong negative correlation was found between TKW and GPC (r = −0.72).

#### 2.4.2. Year and Genetic Structure Influence in TKW and GN

**Figure 4:**
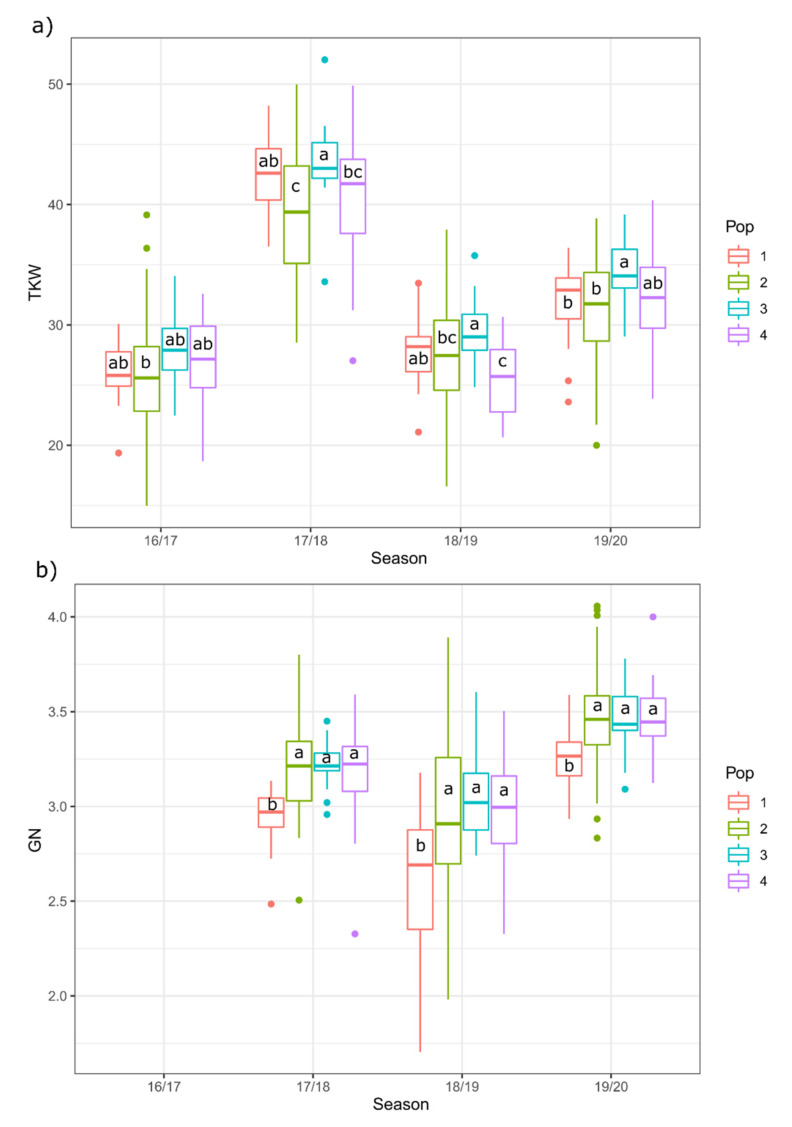
Effect of year and genetic structure of the collection on the (**a**) Thousand Kernel Weight (TKW, g) and (**b**) Grain Number per Spike (GN).

The effect of the year and genetic structure of the collection on the TKW and GN values in the set of landraces was evaluated by an ANOVA test (Figure 4 and Table S3). No year × population interaction effect was detected in any case.

The variability in climatic conditions between years significantly affected TKW, with values being higher in the rainiest season (2017–2018) and being much lower in the driest seasons (2016–2017 and 2018–2019). The genetic structure also had a significant effect on this trait. All populations performed significantly differently across years, and landraces belonging to genetic population 3 were those that achieved higher TKW values in every year. 

For GN, year also had a significant effect, corresponding to the highest values in the mild 2019–2020 season. Genetic populations 2, 3, and 4 showed similar values, with population 1 being the one with the significantly lowest values for any year.

## 3. Discussion

In the present work, genetic variability related to end-use quality was studied in a set of 189 Spanish landraces by characterizing the genes controlling endosperm texture (puroindoline loci) and HMW-Gs composition (*Glu-1* loci), two main quality determinants in bread wheat.

This study of genetic variability in puroindoline genes showed that most landraces had the wild-type soft allele of the *Pina-D1* gene. This allele, originally described by [48], is the most common *Pina-D1* allele among the wheat genotypes [49,50]. The *Pina-D1b* allele, found only in eight of our landraces, was first discovered by [51] and, although extremely rare, has been found in germplasms of diverse origins [32,52,53]. Other rare *Pina-D1* alleles have been described in cultivars from China and India [30,54], but were absent in this collection. Regarding *Pinb-D1*, the *a* allele is normally the most frequent allele in landraces, followed by the *b* and *d* alleles, with *Pinb-D1c* being very rare or even absent [45,55]. This result is opposite to what is frequently found in modern cultivars and breeding lines, where the hard-textured allele *Pinb-D1b* is the most frequent and *Pinb-D1c*, with a similar effect, is also present [55,56,57]. These differences between modern cultivars and landraces are probably caused by the narrow genetic variability in the breeding lines and are the result of a selection pressure commonly attributed to hard-type cultivars. However, in our reference set, which includes mostly cultivars sown in Spain, the allelic distribution is similar to that of landraces, probably due to the fact that, in Spanish breeding programs, hardness has not been included as a breeding target. Three landraces carried a *Pinb-D1* allele that has not been previously described. This allele produced a changed A19T in the predicted protein sequence (see Figure S1). Considering that alanine is an apolar amino acid with a mainly structural role in proteins, this change is likely to be associated with a soft texture, but this prediction should be confirmed in the future by a phenotypic hardness analysis.

Genetic variability for HMW-Gs has been reported to account for up to 60% of the dough strength variation in bread wheat [16]. In the present work, the allelic variations for the *Glu-A1* and *Glu-B1* loci are similar to those found in previous studies on Spanish and Portuguese landraces [42,58]. At the *Glu-A1* locus, the *y* allele has been found at a similar frequency to the *a* and *c* alleles in the landraces analyzed in this work. This allele was first described in lines derived from a Portuguese landrace called Barbela [59], and since then it has been described in very few studies. This may be due to the difficult assignment of this glutenin subunit using only standard SDS–PAGE electrophoresis but could also indicate that it is characteristic of Iberian landraces [42]. Regarding the *Glu-B1* locus, it is worth noting that Spanish landraces present a high frequency of the subunit pairs 20x+20y and 13+16, whereas these combinations are absent in landraces from other regions of the world [60,61,62,63,64], indicating the specific conservation of these alleles in the Iberian Peninsula. The 13+16 pair has also been described at a high frequency (88%) in *Triticum aestivum* ssp. *spelta* from North Spain [58,65]. The HMW glutenins from the *Glu-D1* locus also showed an allelic distribution similar to that found in other previous studies on Iberian landraces [42,58,66] and Chinese landraces, where the 2+12 pair was also the most frequent [60,61], although different from that reported in studies on landraces from other world regions [67,68,69]. Some rare combinations (i.e., 5+12, 2+12*, 12, and 2+10´) appeared at a low frequency. These combinations have been previously described in other germplasm collections [62,63,64], but this is the first time that they have been reported in Iberian landraces, probably due to the large number of and high diversity in the accessions analyzed here.

In this work, the SDSS test was chosen to estimate end-use quality, since it is the most suitable methodology to predict gluten strength when a large number of samples are assessed [70]. In general, the landraces showed lower SDSS values than the reference set but a wider range. This latter finding was expected since modern varieties are quite genetically homogeneous regarding quality due to the small set of beneficial alleles related to this trait selected in breeding. Accordingly, the variability in the *Glu-1* loci observed in the reference set was low and some alleles, such as *Glu-A1b*; *Glu-B1b*, *c*, or *d*; or *Glu-Dd*, were the most frequent (Table S1). The wide range of variability observed in landraces, with some outstanding varieties, indicates the existence of a high degree of genetic variability for this trait that can be explored and further exploited in breeding. 

Due to the strong influence of HMW-Gs on gluten strength, the relation between SDSS values and *Glu-1* variability was analyzed further. At the *Glu-A1* locus, the *a* and *b* alleles have been described as being associated with high end-use quality, with both having a more beneficial effect on gluten strength than the *c* allele [71]. In this work, significant differences in SDSS values were found between the *a* and *b* alleles and between the *b* and *c*, and *b* and *y* alleles, with the *b* allele performing worse than the others. The *y* allele, corresponding to the 2¨ subunit, was also related to high gluten strength, confirming the findings of previous studies [42]. At the *Glu-B1* locus, the *e* allele showed the lowest SDSS values. This result is in accordance with previous studies that have considered it to be a poor end-use quality allele [71]. Significant differences between the *e* allele and the ones with the highest values (*a*, *f*, *i*, *u*, and 7*+9) were found. Among all the *Glu-B1* variants examined, the 7*+9 combination and *i* allele were the most outstanding alleles for gluten strength. These results confirm those obtained by other authors for the 7+9 subunits [23] due to the fact the 7* subunit has been frequently misclassified as a 7 subunit [72]. The 7*+9 combination is present with a high frequency in European modern wheat cultivars, in agreement with its strong relation to high end-use quality [73]. The high SDSS values related to the *f* allele are of particular interest because this allele, as previously mentioned, is characteristic of Iberian landraces [42]. In the present work, a significant influence of the *Glu-D1* genotype was found, showing significant differences in the *a*, *c*, *d*, and *j* alleles. *Glu-D1d* was associated with the highest gluten strength, and *c* and *l* were associated with the lowest. The positive influence of the *Glu-D1d* allele compared to *a* and *c* has also previously been shown [23,71]. Most authors consider the *c* allele to be the one with the lowest influence on the sedimentation volume, but, here, the *l* allele showed even lower SDSS volume values. It should be highlighted that the influence of this allele has not been previously established, as it is a very rare allele. Further studies are required to determine the influence on gluten strength of the new alleles identified in the collection that could not be addressed due to the low number of landraces (only one or two) carrying them (Table 2). 

It is clear that a given *Glu-1* allele can show different effects on gluten properties depending on the genetic background and that specific allelic combinations can have a specific influence on quality [42,43]. For these reasons, *Glu-1* allele combinations were analyzed. Among the thirteen different combinations that were analyzed, *b*:7*+9:*d* showed the highest SDSS values, being significantly different from almost all other allele combinations. This is according to the strong positive effect observed for each of these alleles and in contrast to that observed with the *b*:*u*:*a*, combination, which achieved the second-best SDSS values, but was composed of alleles for which the individual influence was not remarkable. On the other hand, the *a*:*e*:*a*, *b*:*e*:*a*, *b*:*e*:*c*, *c*:*d*:*a*, and *y*:*e*:*a* combinations showed the lowest values. This supports a strong negative influence of the *Glu-B1e* allele, even if some alleles with a positive effect, such as *Glu-A1a* and *Glu-D1a*, are present (Figure 3).

GPC is a parameter that is related to grain quality, but it is not significantly influenced by HMW-Gs subunits composition. We found a negligible correlation between GPC and SDSS (r = 0.074), which is in accordance with some authors [22,64,74], who showed that dough strength is independent of GPC, although the expression of a high-quality genotype requires enough protein content. The genetic components of GPC have been extensively studied in bread wheat [75,76,77,78], but GPC and yield are both largely influenced by environmental conditions (e.g., [79,80,81]). In this work, year had a significant influence on this trait and, as expected, higher GPC content values were observed in the driest seasons [82,83]. However, a higher GPC was consistently observed in the landraces compared to the reference set, in agreement with other studies that have shown that landraces have high GPCs compared to modern varieties, probably due to a dilution effect in the higher-yielding modern cultivars, which may be of particular interest for low-input cropping systems [84,85].

Although breeding for quality must be a key target of a wheat breeding program, yield is a major determinant of crop value and the main goal of any program. The inclusion of landraces in quality breeding programs will be favored if they have good agronomic values. Field characterization can aid breeders when selecting local donor materials that ensure good end-use properties in the derived elite cultivars without high detriment to agronomic performance. In addition, although landraces perform worse than modern varieties in terms of yield, their yield stability can be higher since they are more resilient to adverse conditions and can have a better performance in unfavorable conditions [86,87,88]. It has been proposed that landraces could provide genetic diversity that may contribute to improved yields in rainfed agroecosystems, where heavier kernels may compensate for lower spike fertility [89,90]. 

With this aim, the collection of landraces besides the reference set was evaluated for agronomic performance in four-year trials, in which the yield components TKW and GN were evaluated. TKW is important because it not only determines the flour yield, but also affects the milling quality of wheat grains [91] and because it has been associated with increased grain yield in low- and intermediate-yielding environments [89]. TKW was significantly higher in landraces compared to the reference set in almost every year analyzed and had outstanding values. The water deficit in two of the seasons reduced the TKW values, as reported in other studies [85,92,93]. GN was significantly lower in landraces compared to the reference set, which also agrees with the results of other studies [85]. GN is one of the more stable yield components and is positively correlated with yield, mainly in the driest years [85]. In this work, correlations between traits showed a high and negative correlation between GPC and TKW. A negative correlation between both traits has been found in other studies [64]. Traditionally, high-grain-yielding varieties have been associated with lower-quality parameters, mainly because of the inverse relationship between protein content and grain yield [14,15,94]. Yield is positively associated with GN and TKW, and the two components are usually negatively correlated [37], but, in this work, no correlation was observed between them. 

For TKW and GN, as well as for GPC, the influence of the genetic structure was observed. This supports the existence of different gene pools in the landrace collection that can be explored further in relation to wheat improvement. It is interesting that landraces from population 3 showed high TKW and GPC values, in spite of the negative correlation found between these variables. This group of landraces have a diverse eco-geographical origin, but, with population 4, are the most genetically different from modern cultivars [41]. 

Combining all the traits analyzed, a set of fifteen landraces with high SDSS values and high TKW and GPC values were selected to be characterized further and more deeply in field trials for agronomic performance and for other parameters related to end-use quality (Figure S3 and Table S7). This will allow us to assess their suitability for direct use in farming agrosystems focused on the cultivation of traditional varieties. Interestingly, the landraces selected have very different HMW-Gs compositions (Figure S3 and Table S7), with some of them carrying allele combinations associated with high quality (such as BGE008221) and with some of them carrying poor-quality allele combinations (such as BGE012591). This supports the influence of other genetic factors affecting wheat quality, which have not been evaluated in this work (such as Low-Molecular-Weight Glutenins (LMW-Gs)), and the suitability of this material for association mapping studies aiming to identify new genomic regions that influence the end-use quality of bread wheat. There are other examples of Spanish landraces whose good quality performance is unexpected according to their HMW-Gs genotype. Some of them are used in the elaboration of artisanal baking products, which are currently highly appreciated by consumers [85,95]. 

## 4. Materials and Methods

### 4.1. Plant Material

For this study, a set of 189 bread wheat landraces and old cultivars were selected from the Spanish national collection of *Triticum aestivum* subsp. *vulgare* (Vill.) maintained in Spanish National Plant Genetic Resources Centre (Centro de Recursos Fitogenéticos, CRF-INIA, Alcalá de Henares). All these accessions were homozygous lines derived from GenBank-original accessions, chosen based on their collection site data and agro-morphological characteristics to represent all of the different ecological and geographical areas of Spain [96]. This set was previously analyzed at the genomic level, and the accessions were clustered into four genetic populations, representing different gene pools [41].

Additionally, this study included a set of 18 modern bread wheat varieties composed of the cultivars most widely grown in Spain during the last 50 years. A complete list of the materials is presented in Table S1. Passport and characterization data on each of the entries can be retrieved from the CRF-INIA germplasm database (http://webx.inia.es/web_inventario_nacional/Introduccioneng.asp, accessed on 23 March 2021).

### 4.2. DNA Isolation and Genotyping 

For each accession, genomic DNA were isolated from young leaves of one plant using a modified CTAB method [97].

In order to characterize puroindoline genotypes, the *Pina-D1* and *Pinb-D1* genes were amplified as described in [98]. The PCR-amplified fragments were purified with sepharose columns and sequenced using capillary electrophoresis at Macrogen (Macrogen Europe, Amsterdam, The Netherlands). The sequences were analyzed with Genious version 9.1.8 sequence analysis software [99]. The gene sequences were compared to those available in the GenBank database (*Pina-D1a*: NCBI ID KT885195; *Pinb-D1a*: NCBI ID KT885196; *Pinb-D1b*: NCBI ID KT885197; *Pinb-D1d*: NCBI ID KT885198; and *Pinb-D1ad*: NCBI ID JX187515) and translated to proteins for analysis with the same software.

The total set was also genotyped for the complex *Glu-1* homoeoloci [41], which encodes the HMW-Gs subunits. For this analysis, endosperm proteins were extracted from single seeds according to [100] and fractionated by sodium dodecyl sulfate–polyacrylamide gel electrophoresis (SDS–PAGE) using 12% polyacrylamide gels, as described by [101]. Some subunits (7oe, 7*, and 2¨) were confirmed by PCR analysis [42,72,102]. HMW-Gs allele classification was performed according to the Catalogue of Gene Symbols for Wheat 2013 [46]. 

### 4.3. Grain Quality and Yieldparameters

All landraces were sown for four consecutive years. In the first year (season 2016–2017), the accessions were sown in an augmented design in plots of four rows (1 m long) in Alcala de Henares (40°31’17,8” N, 3°17’33” W, Madrid). In the following years (seasons 2017–2018, 2018–2019, and 2019–2020), landraces and the reference set were sowed in the same conditions in the experimental fields of the School of Agricultural, Food, and Biosystems Engineering (ETSIAAB, Universidad Politécnica de Madrid; 40°25’ N, 3°42’ W). Daily meteorological data were recorded over the period of study (autumn 2016 to summer 2020) at weather stations located near the growing areas. The average monthly precipitation and the minimum and maximum temperatures from October to June are shown in Figure S2 (Supplementary Materials).

The number of Grains per Spike (GN) was recorded according to the International Board of Plant Genetic Resources (IBPGR) from at least five different spikes in each accession. After harvest, Thousand Kernel Weight (TKW) was estimated for all accessions, and a sample of 15 g was ground in a Tekator mill for quality analysis. Protein content on dry matter (GPC) was estimated by near-infrared reflectance analysis (NIR) using a PerCon Inframatic 8600 (Perten Instruments AB, Sweden). Gluten strength was determined on 1 g of whole grain flour samples by an sodium dodecyl sulfate -sedimentation (SDSS) volume test, according to Dick and Quick (1983) [103] with minor modifications. Technical duplicates were used in GPC measurement and the SDSS tests. 

### 4.4. Data Analysis

All statistical analyses were performed with the software R version 3.5.2 [104]. Mean, standard deviation, and maximum and minimum values were calculated for landraces and reference varieties for the four seasons. The Shapiro–Wilk normality test was applied to the four variables: GPC, SDSS, TKW, and GN (p-value < 0.01). For a better fit to normality, a logarithmic transformation was performed for GN before further analysis. For SDSS, no transformation gave a better fit to the normal distribution; therefore, the original values were used in the analysis. A t-test (p-value < 0.05 for GPC, TKW, and GN) or a Wilcox test (p-value < 0.05 for SDSS) was used to compare means between landraces and reference varieties. Spearman correlations between years and variables were calculated (p-value < 0.05). Homocedasticity was checked using the Levene test. The effect of year, the genetic structure of the collection, and their interaction were evaluated by ANOVA for TKW, GN, and GPC (p-value < 0.05). Duncan’s method for multiple mean comparisons was used to study the effect of genetic structure on these variables for each season. For the same purpose and to analyze the relationship between SDSS value and the allelic variation at the *Glu-1* loci, the nonparametric Kruskal–Wallis (p-value < 0.05) and Wilcox tests (p-value < 0.05) were used. Alleles found in less than three landraces were excluded from this analysis.

## 5. Conclusions

In this work, the genetic variability of quality-related genes was evaluated in a set of Spanish wheat landraces. New alleles for puroindolines and HMW-Gs subunits were identified, although the potential of this new variability needs to be studied further by a segregating populations analysis. Moreover, the influence of HMW-Gs variability in gluten strength revealed that some alleles that are not present in modern cultivars, such as *Glu-A1y* and *Glu-B1f*, which is related to Iberian material, may be of particular interest in breeding for quality. The four-year trials allowed us to identify landraces with outstanding values. As this collection has been previously throughput genotyped [41], these data in combination with the results obtained in this work can be very valuable in identifying genomic regions associated with quality- and yield-related traits using Genome Wide Association Studies (GWAS). In addition, several interesting landraces that showed high gluten strength and high GPC will be characterized more deeply in field trials to assess their suitability for organic cropping systems, where yield is not as important as the quality-added value provided. 

## Figures and Tables

**Table 1:** Frequency of the different *Pina-D1*/*Pinb-D1* alleles observed in the bread wheat landraces and the analyzed reference set. Endosperm texture associated with previous described alleles is also shown [27,45].

	Landraces						Reference Set			
*pinA-D1* allele	*a*	*a*	*a*	*a*	*a*	*b*	*a*	*a*	*a*	*b*
*pinB-D1* allele	*a*	*b*	*d*	*new1 (ad)*	*new2*	*a*	*a*	*b*	*d*	*a*
Endosperm texture	soft	hard	hard	hard	n.d.	hard	soft	hard	hard	hard
N	150	5	5	15	3	8	8	3	3	3
%	80.65	2.69	2.69	8.06	1.61	4.30	47.06	17.65	17.65	17.65

n.d.: not described.

**Table 2:** Frequency of the different high-molecular-weight glutenin subunits (HMW-GS) observed for *Glu-1* loci in the 189 bread wheat landraces analyzed. The allele correspondence is indicated for the variants that have already been designated.

Locus	Allele	HMW-GS	N	%
*Glu-A1*	*a*	1	31	16.40
	*b*	2*	101	53.44
	*c*	null	22	11.64
	*y*	2··	34	17.99
	*New1*	-	1	0.53
*Glu-B1*	*a*	7	6	3.17
	*d*	6+8	11	5.82
	*e*	20x+20y	89	47.09
	*f*	13+16	30	15.87
	*aq*	32+33	6	3.17
	7*+9	7*+9	7	3.70
	*al*	7oe+8	3	1.59
	*am*	18	4	2.12
	*i*	17+18	3	1.59
	*u*	7*+8	26	13.76
	*New2*	-	1	0.53
	*New3*	-	1	0.53
	*New4*	-	1	0.53
	*New5*	-	1	0.53
*Glu-D1*	*a*	2+12	127	67.20
	*c*	4+12	44	23.28
	*d*	5+10	8	4.23
	*h*	5+12	1	0.53
	*j*	2+12*	2	1.06
	*l*	12	3	1.59
	*u*	2+10’	1	0.53
	*New6*	-	2	1.06
	*New7*	-	1	0.53

**Table 3:** Mean, range, and standard deviation for Grain Protein Content (GPC) and SDS Sedimentation (SDSS) test values.

			Mean	Min	Max	sd	*p*-Value Ld/Ref ^#^
GPC (%)	2016/2017	Ld	17.59	13.97	20.96	1.24	-
		Ref	-	-	-	-	
	2017/2018	Ld	11.51	8.97	15.44	1.16	***
		Ref	9.28	7.84	10.86	0.76	
	2018/2019	Ld	17.05	13.41	20.33	1.19	***
		Ref	15.30	12.96	16.96	1.15	
	2019/2020	Ld	14.47	11.23	18.78	1.60	***
		Ref	13.23	11.77	15.05	0.95	
SDSS (mm)	2016/2017	Ld	55.45	23.50	115.50	23.28	-
		Ref	-	-	-	-	
	2017/2018	Ld	37.90	18	111	14.78	***
		Ref	52.28	34	69	10.44	
	2018/2019	Ld	41.51	10	93.50	17.02	***
		Ref	87.89	58.5	112	16.09	
	2019/2020	Ld	58.14	18.50	107.50	20.39	***
		Ref	83.22	66	97.5	10.89	

-: data not available. ^#^: * 0.05 > *p* > 0.05; ** 0.05 > *p* > 0.005; *** *p* < 0.005; ns: nonsignificant. Ld: landraces; Ref: reference set.

**Table 4:** Mean, range, and standard deviation for Thousand Kernel Weight (TKW) and Grain Number per Spike (GN).

			Mean	Min	Max	sd	*p*-Value Ld/Ref ^#^
TKW (g)	2016/2017	Ld	26.20	14.98	39.14	3.99	-
		Ref	-	-	-	-	
	2017/2018	Ld	40.24	27.03	52.03	5.01	***
		Ref	35.11	25.73	43.71	5.13	
	2018/2019	Ld	27.19	16.59	37.92	3.84	ns
		Ref	27.42	22.37	33.31	3.09	
	2019/2020	Ld	31.92	20.00	40.36	4.02	***
		Ref	29.84	22.08	35.60	3.87	
GN	2016/2017	Ld	-	-	-	-	-
		Ref	-	-	-	-	
	2017/2018	Ld	24.67	10.25	44.75	6.31	***
		Ref	36.36	24.5	52.25	7.04	
	2018/2019	Ld	19.91	5.5	49.00	7.61	***
		Ref	40.16	18	57.75	8.99	
	2019/2020	Ld	31.76	17.00	57.80	7.24	***
		Ref	46.46	29.4	62.8	8.69	

-: data not available. ^#^: * 0.05 > *p* > 0.05; ** 0.05 > *p* > 0.005; *** *p* < 0.005; ns: nonsignificant. Ld: landraces; Ref: reference set.

## Data Availability

Data is contained within the article or Supplementary Materials.

## Supplementary Materials

The following are available online at https://www.mdpi.com/2223-7747/10/4/620/s1, Table S1. Accessions genetic profile and data from the field trials. Figure S1. Alignment of the puroindoline b predicted protein sequence for each allele: the polymorphism characteristic of each allele is highlighted. Figure S2. Monthly rainfall (mm) and mean maximum (Tmax, T ºC) and minimum (Tmin, T ºC) temperature recorded from October to June at the field trial sites during (a) 2016–2017, (b) 2017–2018, (c) 2018–2019, and (d) 2019–2020. Table S2. Spearman’s correlation among (A) traits and (B) years for Thousand Kernel Weight (TKW), Grain Protein Content (GPC), Grain Number (GN), and SDS Sedimentation (SDSS) test. Table S3. Mean, standard deviation, and F values for the effect of the *Glu-1* loci on the SDS Sedimentation (SDSS) test values (mm). Table S4. Wilcox test for the *Glu-A1*, *Glu-B1*, and *Glu-D1* alleles. Table S5. Mean and standard deviation of SDS Sedimentation test (mm) values for *Glu-1* allele combinations. Table S6. Wilcox test for allele combination *Glu-A1*:*Glu-B1*:*Glu-D1*. Table S7. Mean and standard deviation for SDS Sedimentation (SDSS) test (mm), Grain Protein Content (GPC, %), and Thousand Kernel Weight (TKW, g) for a selected set of landraces and their HMW-Gs compositions. Figure S3. Set of fifteen landraces selected (in red) based on SDS Sedimentation (SDSS) test values (mm), Thousand Kernel Weight (TKW, g) and Grain Protein Content (GPC, %) to be further and deeply characterized in field trials.

## References

[1] Food and Agriculture Organization of the United Nations FAOSTAT. http://www.fao:faostat/es/#data/QC..

[2] Marcussen T., Sandve S.R., Heier L., Spannagl M., Pfeifer M., Jakobsen K.S., Wulff B.B., Steuernagel B., Mayer K.F., Olsen O. (2014). Ancient hybridizations among the ancestral genomes of bread wheat. Science.

[3] Shewry P.R., Hey S.J. (2015). The contribution of wheat to human diet and health. Food Energy Secur..

[4] Cox T.S. (1997). Deepening the wheat gene pool. J. Crop Prod..

[5] Ficiciyan A., Loos J., Sievers-Glotzbach S., Tscharntke T. (2018). More than yield: Ecosystem services of traditional versus modern crop varieties revisited. Sustainability.

[6] Alipour H., Bihamta M.R., Mohammadi V., Peyghambari S.A., Bai G., Zhang G. (2017). Genotyping-by-sequencing (GBS) revealed molecular genetic diversity of Iranian wheat landraces and cultivars. Front. Plant. Sci..

[7] Nazco R., Villegas D., Ammar K., Pena R.J., Moragues M., Royo C. (2012). Can Mediterranean durum wheat landraces contribute to improved grain quality attributes in modern cultivars?. Euphytica.

[8] Wingen L.U., Orford S., Goram R., Leverington-Waite M., Bilham L., Patsiou T.S., Ambrose M., Dicks J., Griffiths S. (2014). Establishing the AE Watkins landrace cultivar collection as a resource for systematic gene discovery in bread wheat. Theor. Appl. Genet..

[9] Jordan K.W., Wang S., Lun Y., Gardiner L., MacLachlan R., Hucl P., Wiebe K., Wong D., Forrest K.L., Sharpe A.G. (2015). A haplotype map of allohexaploid wheat reveals distinct patterns of selection on homoeologous genomes. Genome Biol..

[10] Sehgal D., Vikram P., Sansaloni C.P., Ortiz C., Saint Pierre C., Payne T., Ellis M., Amri A., Petroli C.D., Wenzl P. (2015). Exploring and mobilizing the gene bank biodiversity for wheat improvement. PLoS ONE.

[11] Riaz A., Hathorn A., Dinglasan E., Ziems L., Richard C., Singh D., Mitrofanova O., Afanasenko O., Aitken E., Godwin I. (2017). Into the vault of the Vavilov wheats: Old diversity for new alleles. Genet. Resour. Crop Evol..

[12] Asseng S., Martre P., Maiorano A., Rötter R.P., O’Leary G.J., Fitzgerald G.J., Girousse C., Motzo R., Giunta F., Babar M.A. (2019). Climate change impact and adaptation for wheat protein. Global Chang. Biol..

[13] Groos C., Robert N., Bervas E., Charmet G. (2003). Genetic analysis of grain protein-content, grain yield and thousand-kernel weight in bread wheat. Theor. Appl. Genet..

[14] Brancourt-Hulmel M., Doussinault G., Lecomte C., Bérard P., Le Buanec B., Trottet M. (2003). Genetic improvement of agronomic traits of winter wheat cultivars released in France from 1946 to 1992. Crop Sci..

[15] De Vita P., Matteu L., Mastrangelo A.M., Di Fonzo N., Cattivelli L. (2007). Effects of breeding activity on durum wheat traits breed in Italy during the 20th century. Ital. J. Agron..

[16] Payne P.I., Nightingale M.A., Krattiger A.F., Holt L.M. (1987). The relationship between HMW glutenin subunit composition and the bread-making quality of British-grown wheat varieties. J. Sci. Food Agric..

[17] Shewry P.R., Halford N.G., Tatham A.S. (1992). High molecular weight subunits of wheat glutenin. J. Cereal Sci..

[18] Shewry P.R., Halford N.G., Lafiandra D. (2003). Genetics of Wheat Gluten Proteins. Adv. Genet..

[19] Payne P.I., Holt L.M., Law C.N. (1981). Structural and genetical studies on the high-molecular-weight subunits of wheat glutenin. Theor. Appl. Genet..

[20] Pogna N.E., Borghi B., Mellini F., Peruffo A.D.B., Nash R.J. (1986). Electrophoresis of gliadins for estimating the genetic purity in bread wheat seed production. Genet. Agr..

[21] Branlard G., Pierre J., Rousset M. (1992). Selection indices for quality evaluation in wheat breeding. Theor. Appl. Genet..

[22] Branlard G., Dardevet M. (1985). Diversity of grain protein and bread wheat quality: II. Correlation between high molecular weight subunits of glutenin and flour quality characteristics. J. Cereal Sci..

[23] Pirozi M.R., Margiotta B., Lafiandra D., MacRitchie F. (2008). Composition of polymeric proteins and bread-making quality of wheat lines with allelic HMW-GS differing in number of cysteines. J. Cereal Sci..

[24] Martin J.M., Frohberg R.C., Morris C.F., Talbert L.E., Giroux M.J. (2001). Milling and bread baking traits associated with puroindoline sequence type in hard red spring wheat. Crop Sci..

[25] Morris C.F., DeMacon V.L., Giroux M.J. (1999). Wheat grain hardness among chromosome 5D homozygous recombinant substitution lines using different methods of measurement. Cereal Chem..

[26] Morris C.F., Rose S.P. (1996). Wheat. Cereal Grain Quality.

[27] Morris C.F. (2002). Puroindolines: The molecular genetic basis of wheat grain hardness. Plant Mol. Biol..

[28] Bhave M., Morris C.F. (2008). Molecular genetics of puroindolines and related genes: Allelic diversity in wheat and other grasses. Plant Mol. Biol..

[29] Ma X., Sajjad M., Wang J., Yang W., Sun J., Li X., Zhang A., Liu D. (2017). Diversity, distribution of Puroindoline genes and their effect on kernel hardness in a diverse panel of Chinese wheat germplasm. BMC Plant Biol..

[30] Kumar R., Arora S., Singh K., Garg M. (2015). Puroindoline allelic diversity in Indian wheat germplasm and identification of new allelic variants. Breed. Sci..

[31] Wang J., Sun J., Liu D., Yang W., Wang D., Tong Y., Zhang A. (2008). Analysis of *Pina* and *Pinb* alleles in the micro-core collections of Chinese wheat germplasm by Ecotilling and identification of a novel *Pinb* allele. J. Cereal Sci..

[32] Lillemo M., Chen F., Xia X., William M., Peña R.J., Trethowan R., He Z. (2006). Puroindoline grain hardness alleles in CIMMYT bread wheat germplasm. J. Cereal Sci..

[33] Ayala M., Guzmán C., Alvarez J.B., Peña R.J. (2013). Characterization of genetic diversity of puroindoline genes in Mexican wheat landraces. Euphytica.

[34] Brown A. (1989). Core collections: A practical approach to genetic resources management. Genome.

[35] Rharrabti Y., Villegas D., Royo C., Martos-Núñez V., Del Moral L.G. (2003). Durum wheat quality in Mediterranean environments: II. Influence of climatic variables and relationships between quality parameters. Field Crops Res..

[36] Sehgal D., Autrique E., Singh R., Ellis M., Singh S., Dreisigacker S. (2017). Identification of genomic regions for grain yield and yield stability and their epistatic interactions. Sci. Rep..

[37] Sukumaran S., Lopes M., Dreisigacker S., Reynolds M. (2018). Genetic analysis of multi-environmental spring wheat trials identifies genomic regions for locus-specific trade-offs for grain weight and grain number. Theor. Appl. Genet..

[38] Sanchez-Garcia M., Álvaro F., Martín-Sánchez J.A., Sillero J.C., Escribano J., Royo C. (2012). Breeding effects on the genotype× environment interaction for yield of bread wheat grown in Spain during the 20th century. Field Crops Res..

[39] Ruiz M., Giraldo P., Royo C., Villegas D., Aranzana M.J., Carrillo J.M. (2012). Diversity and genetic structure of a collection of Spanish durum wheat landraces. Crop Sci..

[40] Giraldo P., Royo C., González M., Carrillo J.M., Ruiz M. (2016). Genetic diversity and association mapping for agromorphological and grain quality traits of a structured collection of durum wheat landraces including subsp. *durum*, *turgidum* and *diccocon*. PLoS ONE.

[41] Pascual L., Ruiz M., López-Fernández M., Pérez-Peña H., Benavente E., Vázquez J.F., Sansaloni C., Giraldo P. (2020). Genomic analysis of Spanish wheat landraces reveals their variability and potential for breeding. BMC Genom..

[42] Giraldo P., Rodríguez-Quijano M., Simon C., Vázquez J.F., Carrillo J.M. (2010). Allelic variation in HMW glutenins in Spanish wheat landraces and their relationship with bread quality. Span. J. Agric. Res..

[43] Chacón E.A., Vázquez F.J., Giraldo P., Carrillo J.M., Benavente E., Rodríguez-Quijano M. (2020). Allelic variation for prolamins in Spanish durum wheat landraces and its relationship with quality traits. Agronomy.

[44] Martos V., Royo C., Rharrabti Y., Del Moral L.G. (2005). Using AFLPs to determine phylogenetic relationships and genetic erosion in durum wheat cultivars released in Italy and Spain throughout the 20th century. Field Crops Res..

[45] Ayala M., Guzmán C., Peña R.J., Alvarez J.B. (2016). Genetic diversity and molecular characterization of puroindoline genes (*Pina-D1* and *Pinb-D1*) in bread wheat landraces from Andalusia (Southern Spain). J. Cereal Sci..

[46] McIntosh R.A., Dubcovsky J., Rogers W.J., Morris C., Appels R., Xia X.C. Catalogue of gene symbols for wheat: 2013–2014. Proceedings of the 12th International Wheat Genetics Symposium.

[47] García A.G. (1999). Cultivos Herbáceos Extensivos.

[48] Gautier M., Aleman M., Guirao A., Marion D., Joudrier P. (1994). *Triticum aestivum* puroindolines, two basic cystine-rich seed proteins: cDNA sequence analysis and developmental gene expression. Plant Mol. Biol..

[49] Iftikhar A., Ali I. (2017). Kernel softness in wheat is determined by starch granule bound Puroindoline proteins. J. Plant Biochem. Biotechnol..

[50] Nadolska-Orczyk A., Gasparis S., Orczyk W. (2009). The determinants of grain texture in cereals. J. Appl. Genet..

[51] Giroux M.J., Morris C.F. (1998). Wheat grain hardness results from highly conserved mutations in the friabilin components puroindoline a and b. Proc. Natl. Acad. Sci USA.

[52] Chen F., Li H., Li X., Dong Z., Zuo A., Shang X., Cui D. (2013). Alveograph and Mixolab parameters associated with *Puroindoline-D1* genes in Chinese winter wheats. J. Sci. Food Agric..

[53] Eagles H.A., Cane K., Eastwood R.F., Hollamby G.J., Kuchel H., Martin P.J., Cornish G.B. (2006). Contributions of glutenin and puroindoline genes to grain quality traits in southern Australian wheat breeding programs. Aust. J. Agric. Res..

[54] Chang C., Zhang H., Xu J., Li W., Liu G., You M., Li B. (2006). Identification of allelic variations of puroindoline genes controlling grain hardness in wheat using a modified denaturing PAGE. Euphytica.

[55] Przyborowski M., Gasparis S., Kała M., Orczyk W., Nadolska-Orczyk A. (2020). The variability of puroindoline-encoding alleles and their influence on grain hardness in modern wheat cultivars cultivated in Poland, breeding lines and Polish old landraces (*Triticum aestivum* L.). Agronomy.

[56] Lillemo M., Morris C.F. (2000). A leucine to proline mutation in puroindoline b is frequently present in hard wheats from Northern Europe. Theor. Appl. Genet..

[57] Huang X., Röder M.S. (2005). Development of SNP assays for genotyping the Puroindoline b gene for grain hardness in wheat using pyrosequencing. J. Agric. Food Chem..

[58] Rodríguez-Quijano M., Vázquez J.F., Carrillo J.M. (1990). Variation of high molecular weight glutenin subunits in Spanish landraces of *Triticum aestivum* ssp. vulgare and ssp. spelta. J. Genet. Breed..

[59] Igrejas G., Carnide V., Guedes Pinto H., Branlard G., Gateau I. (1997). Storage protein diversity within the old Portuguese bread wheat Barbela population [*Triticum aestivum*]. J. Genet. Breed..

[60] Fang J., Liu Y., Luo J., Wang Y., Shewry P.R., He G. (2009). Allelic variation and genetic diversity of high molecular weight glutenin subunit in Chinese endemic wheats (*Triticum aestivum* L.). Euphytica.

[61] Li Y., Huang C., Sui X., Fan Q., Li G., Chu X. (2009). Genetic variation of wheat glutenin subunits between landraces and varieties and their contributions to wheat quality improvement in China. Euphytica.

[62] Goel S., Yadav M., Singh K., Jaat R.S., Singh N.K. (2018). Exploring diverse wheat germplasm for novel alleles in HMW-GS for bread quality improvement. J. Food Sci. Technol..

[63] Dai S., Xu D., Yan Y., Wen Z., Zhang J., Chen H., Lu Z., Li H., Cong H., Wei Y. (2020). Characterization of high-and low-molecular-weight glutenin subunits from Chinese Xinjiang wheat landraces and historical varieties. J. Food Sci. Technol..

[64] Maryami Z., Azimi M.R., Guzman C., Dreisigacker S., Najafian G. (2020). Puroindoline (*Pina-D1* and *Pinb-D1*) and waxy (*Wx-1*) genes in Iranian bread wheat (*Triticum aestivum* L.) landraces. Biotechnol. Biotechnol. Equip..

[65] Caballero L., Martin L.M., Alvarez J.B. (2004). Intra-and interpopulation diversity for HMW glutenin subunits in Spanish spelt wheat. Genet. Resour. Crop Evol..

[66] Brites C., Bagulho A.S., Rodriguez-Quijano M., Carrillo J.M. (2000). Effects of HMW glutenin subunits on some quality parameters of Portuguese landraces of *Triticum aestivum* ssp. *vulgare*. Wheat Gluten, Proceedings of the 7th International Workshop Gluten 2000, Bristol, UK, 2–6 April 2000.

[67] Payne P.I., Lawrence G.J. (1983). Catalogue of alleles for the complex gene loci, *Glu-A1*, *Glu-B1*, and *Glu-D1* which code for high-molecular-weight subunits of glutenin in hexaploid wheat. Cereal Res. Commun..

[68] Morgunov A.I., Pena R.J., Crossa J., Rajaram S. (1993). Worldwide distribution of *Glu-1* alleles in bread wheat. J. Genet. Breed..

[69] Tohver M. (2007). High molecular weight (HMW) glutenin subunit composition of some Nordic and Middle European wheats. Genet. Resour. Crop Evol..

[70] Morris C.F., Paszczynska B., Bettge A.D., King G.E. (2007). A critical examination of the sodium dodecyl sulfate (SDS) sedimentation test for wheat meals. J. Sci. Food Agric..

[71] Cornish G., Békés F., Eagles H., Payne P. (2006). Prediction of dough properties for bread wheats. Gliadin and Glutenin: The Unique Balance of Wheat Quality.

[72] Espí A., Giraldo P., Rodriguez-Quijano M., Carrillo J.M. (2012). A PCR-based method for discriminating between high molecular weight glutenin subunits Bx7 and Bx7* in *Triticum aestivum* L.. Plant Breed..

[73] Nucia A., Okoń S., Tomczyńska-Mleko M. (2019). Characterization of HMW glutenin subunits in European spring common wheat (*Triticum aestivum* L.). Genet. Resour. Crop Evol..

[74] Brunori A., Galterio G., Zannettino C., Pogna N.E. (1989). Bread-Making Quality indices in *Triticum aestivum* orogenies. Implications in breeding for better bread wheat. Plant Breed..

[75] Joppa L.R., Du C., Hart G.E., Hareland G.A. (1997). Mapping gene (s) for grain protein in tetraploid wheat (*Triticum turgidum* L.) using a population of recombinant inbred chromosome lines. Crop Sci..

[76] Prasad M., Varshney R.K., Kumar A., Balyan H.S., Sharma P.C., Edwards K.J., Dhaliwal H.S., Roy J.K., Gupta P.K. (1999). A microsatellite marker associated with a QTL for grain protein content on chromosome arm 2DL of bread wheat. Theor. Appl. Genet..

[77] Perretant M.R., Cadalen T., Charmet G., Sourdille P., Nicolas P., Boeuf C., Tixier M.H., Branlard G., Bernard S. (2000). QTL analysis of bread-making quality in wheat using a doubled haploid population. Theor. Appl. Genet..

[78] Zanetti S., Winzeler M., Feuillet C., Keller B., Messmer M. (2001). Genetic analysis of bread-making quality in wheat and spelt. Plant Breed..

[79] Bhullar S.S., Jenner C.F. (1985). Differential responses to high temperatures of starch and nitrogen accumulation in the grain of four cultivars of wheat. Funct. Plant Biol..

[80] Wardlaw I.F., Wrigley C.W. (1994). Heat tolerance in temperate cereals: An overview. Funct. Plant Biol..

[81] Daniel C., Triboi E. (2000). Effects of temperature and nitrogen nutrition on the grain composition of winter wheat: Effects on gliadin content and composition. J. Cereal Sci..

[82] López-Bellido L., Fuentes M., Castillo J.E., López-Garrido F.J. (1998). Effects of tillage, crop rotation and nitrogen fertilization on wheat-grain quality grown under rainfed Mediterranean conditions. Field Crops Res..

[83] Gürsoy S., Sessiz A., Malhi S.S. (2010). Short-term effects of tillage and residue management following cotton on grain yield and quality of wheat. Field Crops Res..

[84] Dotlačil L., Hermuth J., Stehno Z., Dvořáček V., Bradová J., Leišová L. (2010). How can wheat landraces contribute to present breeding. Czech J. Genet. Plant Breed..

[85] Ruiz M., Zambrana E., Fite R., Sole A., Tenorio J.L., Benavente E. (2019). Yield and quality performance of traditional and improved bread and durum wheat varieties under two conservation tillage systems. Sustainability.

[86] Zeven A.C. (1998). Landraces: A review of definitions and classifications. Euphytica.

[87] Kyzeridis N., Biesantz A., Limberg P. (1995). Comparative trials with durum-wheat landraces and cultivars in different ecological environments in the Mediterranean region. J. Agron. Crop. Sci..

[88] Carranza-Gallego G., Guzmán G.I., Garcia-Ruiz R., Gonzalez de Molina M., Aguilera E. (2019). Addressing the role of landraces in the sustainability of Mediterranean agroecosystems. Sustainability.

[89] Lopes M.S., Reynolds M.P., Manes Y., Singh R.P., Crossa J., Braun H.J. (2012). Genetic yield gains and changes in associated traits of CIMMYT spring bread wheat in a “historic” set representing 30 years of breeding. Crop Sci..

[90] Lopes M.S., El-Basyoni I., Baenziger P.S., Singh S., Royo C., Ozbek K., Aktas H., Ozer E., Ozdemir F., Manickavelu A. (2015). Exploiting genetic diversity from landraces in wheat breeding for adaptation to climate change. J. Exp. Bot..

[91] Campbell K.G., Bergman C.J., Gualberto D.G., Anderson J.A., Giroux M.J., Hareland G., Fulcher R.G., Sorrells M.E., Finney P.L. (1999). Quantitative trait loci associated with kernel traits in a soft× hard wheat cross. Crop Sci..

[92] Royo C., Abaza M., Blanco R., del Moral L.F.G. (2000). Triticale grain growth and morphometry as affected by drought stress, late sowing and simulated drought stress. Funct. Plant Biol..

[93] Diacono M., Castrignanò A., Troccoli A., De Benedetto D., Basso B., Rubino P. (2012). Spatial and temporal variability of wheat grain yield and quality in a Mediterranean environment: A multivariate geostatistical approach. Field Crops Res..

[94] Austin R.B., Bingham J., Blackwell R.D., Evans L.T., Ford M.A., Morgan C.L., Taylor M. (1980). Genetic improvements in winter wheat yields since 1900 and associated physiological changes. J. Agric. Sci..

[95] Cerere Project. http://cere2020.eu/ptoject/.

[96] Pascual L., Fernández M., Aparicio N., López-Fernández M., Fité R., Giraldo P., Ruiz M. (2020). Development of a multipurpose core collection of bread wheat based on high-throughput genotyping data. Agronomy.

[97] Doyle J.J., Doyle J.L. (1987). A rapid DNA isolation procedure for small quantities of fresh leaf tissue. Phytochem. Bull..

[98] Ribeiro M., Rodríguez-Quijano M., Giraldo P., Pinto L., Vázquez J.F., Carrillo J.M., Igrejas G. (2017). Effect of allelic variation at glutenin and puroindoline loci on bread-making quality: Favorable combinations occur in less toxic varieties of wheat for celiac patients. Eur. Food Res. Technol..

[99] (2017). Geneious.

[100] Singh N.K., Shepherd K.W., Cornish G.B. (1991). A simplified SDS-page procedure for separating LMW subunits of glutenin. J Cereal Sci..

[101] Payne P.I., Law C.N., Mudd E.E. (1980). Control by homoeologous group 1 chromosomes of the high-molecular-weight subunits of glutenin, a major protein of wheat endosperm. Theor. Appl. Genet..

[102] Ragupathy R., Naeem H.A., Reimer E., Lukow O.M., Sapirstein H.D., Cloutier S. (2008). Evolutionary origin of the segmental duplication encompassing the wheat *GLU-B1* locus encoding the overexpressed Bx7 (Bx7 OE) high molecular weight glutenin subunit. Theor. Appl. Genet..

[103] Dick J.W., Quick J.S. (1983). A Modified Screening Test for Rapid Estimation of Gluten Strength in Early-Generation Durum Wheat Breeding Lines. Cereal Chem..

[104] R Core Team (2018). R: A language and Environment for Statistical Computing.

