# Supplementary material for: Exploring the End-Use Quality Potential of a Collection of Spanish Bread Wheat Landraces
Source: Plants (Basel). 2021 Mar 24;10(4):620. doi: 10.3390/plants10040620 (PMC8064353; doi:10.3390/plants10040620)
Supplement: Supplementary file 1 [file plants-10-00620-s001.zip › SUP/Supplementary figures S1-S3 and tables S2-S7_R1.pdf]

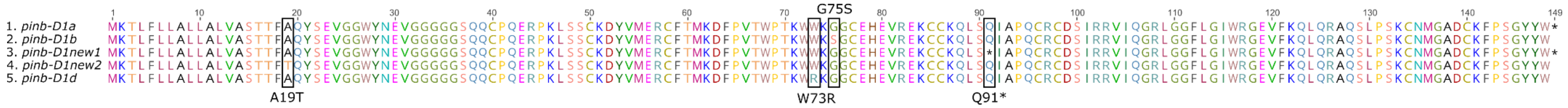

Figure S1. Alignment of Puroindoline b predicted protein sequence for each allele. Polymorphism characteristic of each allele is highlighted.

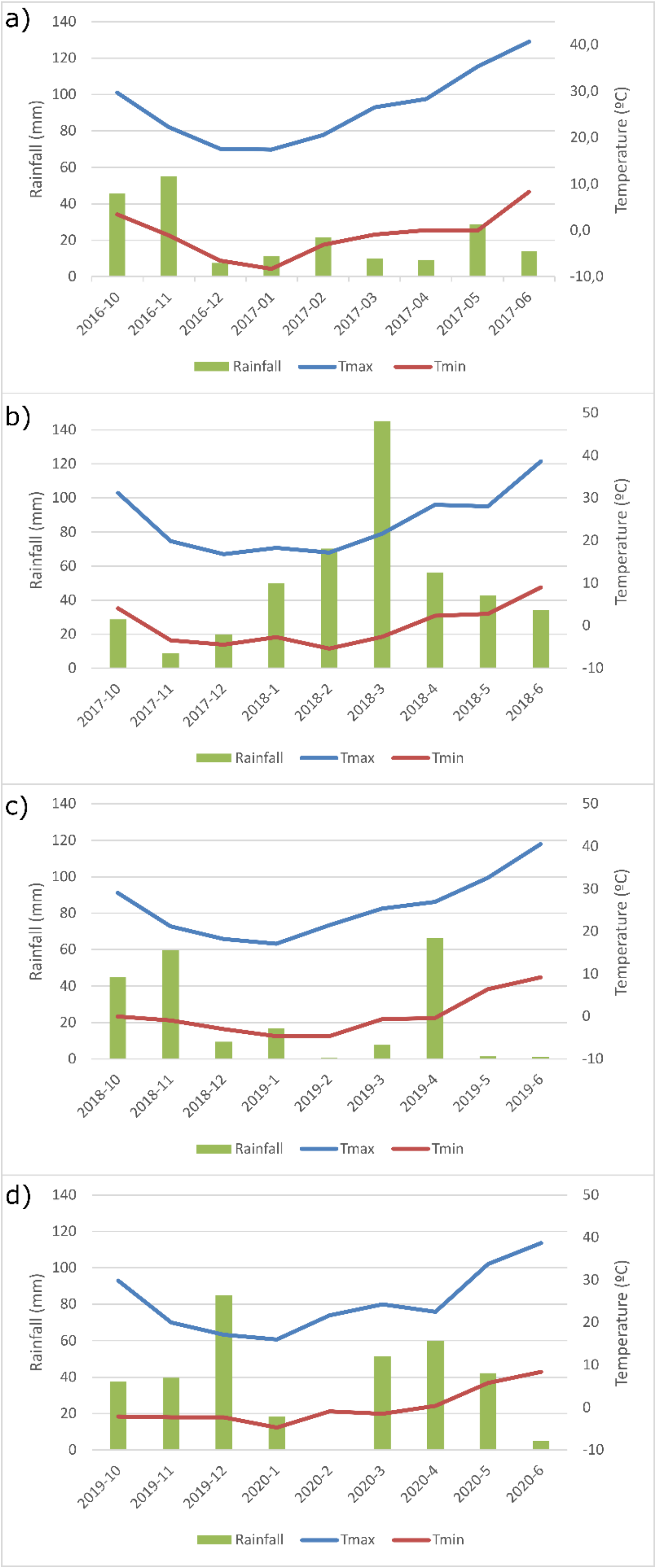

Figure S2. Monthly rainfall (mm) and mean maximum (Tmax, TºC) and minimum (Tmin, TºC) temperature recorded from October to June at the field trial sites during (a) 2016-2017, (b) 2017-2018, (c) 2018-2019 and (d) 2019-2020.

Table S2. Spearman's correlation among (A) traits, and (B) years for Thousand Kernel Weight (TKW), Grain Protein Content (GPC), Grain Number (GN) and SDS Sedimentation test (SDSS)

(A)

	GPC	SDSS	TKW	GN
**GPC**	1			
**SDSS**	0.074	1		
**TKW**	-0.716	-0.225	1	
**GN**	-0.255	0.319	0.098	1

(B)

GPC	2016/2017	2017/2018	2018/2019	2019/2020
**2016/2017**	1			
**2017/2018**	0.435	1		
**2018/2019**	0.468	0.359	1	
**2019/2020**	0.446	0.337	0.577	1

SDSS	2016/2017	2017/2018	2018/2019	2019/2020
**2016/2017**	1			
**2017/2018**	0.739	1		
**2018/2019**	0.777	0.645	1	
**2019/2020**	0.846	0.762	0.791	1

TKW	2016/2017	2017/2018	2018/2019	2019/2020
**2016/2017**	1			
**2017/2018**	0.501	1		
**2018/2019**	0.430	0.354	1	
**2019/2020**	0.568	0.569	0.475	1

GN	2016/2017	2017/2018	2018/2019	2019/2020
**2016/2017**	1			
**2017/2018**	-	1		
**2018/2019**	-	0.395	1	
**2019/2020**	-	0.472	0.431	1

Table S3. Mean, standard deviation and F values for the effect of the *Glu-1* loci on the SDS Sedimentation test values (SDSS, mm)

	**allele**	**SDSS (mm)**		**F value**
		mean	sd	
Glu-A1	*a*	52.14	± 22.00	***
	b	45.63	± 21.52	
	c	50.91	± 21.10	
	y	50.61	± 17.11	
Glu-B1	7*+9	71.93	± 25.91	***
	a	54.23	± 17.39	
	al	53.50	± 25.47	
	am	46.17	± 12.05	
	aq	49.77	± 19.65	
	d	44.70	± 14.52	
	e	39.84	± 16.99	
	f	57.92	± 20.13	
	i	77.00	± 22.09	
	u	56.71	± 21.58	
Glu-D1	*a*	50.60	± 20.06	***
	c	39.09	± 18.69	
	d	68.44	± 28.58	
	l	35.75	± 12.65	

* 0.05 > p > 0.05; ** 0.05 > p > 0.005; *** p < 0.005; ns: non significant.

Table S4. Wilcox test for *Glu-A1*, *Glu-B1* and *Glu-D1* alleles

Glu-A1	*a*	*b*	*c*	*y*
a	-			
b	**	-		
c	ns	*	-	
y	ns	***	ns	-

Glu-B1	7*+9	*a*	*al*	*am*	*aq*	*d*	*e*	*f*	*i*	*u*
7*+9	-									
a	ns	-								
al	ns	ns	-							
am	*	ns	ns	-						
aq	ns	ns	ns	ns	-					
d	***	ns	ns	ns	ns	-				
e	***	**	ns	ns	ns	ns	-			
f	ns	ns	ns	ns	ns	*	***	-		
i	ns	ns	ns	*	*	**	***	ns	-	
u	ns	ns	ns	ns	ns	ns	***	ns	ns	-

Glu-D1	*a*	*c*	*d*	*l*
a	-			
c	***	-		
d	**	***	-	
l	*	ns	**	-

* 0.05 > p > 0.05; ** 0.05 > p > 0.005; *** p < 0.005; ns: non significant.

Table S5. Mean and standard deviation of SDS Sedimentation test (mm) values for *Glu-1* alleles' combination

Allele's combination	N	mean	sd
a:*d*:*a*	5	45.28	± 12.06
a:*e*:*a*	10	40.39	± 17.79
b:7*+9:*d*	3	90.21	± 24.57
b:*a*:*a*	4	51.06	± 17.08
b:*e*:*a*	37	43.93	± 18.37
b:*e*:*c*	29	31.58	± 9.83
b:*f*:*a*	5	58.48	± 17.76
b:*u*:*a*	7	64.36	± 19.49
b:*u*:*d*	3	46.75	± 16.15
c:*d*:*a*	3	38.50	± 8.28
c:*u*:*a*	14	47.11	± 19.32
y:*e*:*a*	4	40.97	± 14.53
y:*f*:*a*	19	56.90	± 17.68

Table S6. Wilcox test for alleles' combination G*lu-A1*:*Glu-B1*:*Glu-D1*

	a:d:a	*a:e:a*	*b:7*+9:d*	*b:a:a*	*b:e:a*	*b:e:c*	*b:f:a*	*b:u:a*	*b:u:d*	*c:d:a*	*c:u:a*	*y:e:a*	*y:f:a*
a:d:a	-												
a:e:a	ns	-											
b:7*+9:d	**	***	-										
b:a:a	ns	ns	*	-									
b:e:a	ns	ns	***	ns	-								
b:e:c	***	ns	***	***	***	-							
b:f:a	ns	*	ns	ns	*	***	-						
b:u:a	*	***	ns	ns	***	***	ns	-					
b:u:d	ns	ns	ns	ns	ns	ns	ns	ns	-				
c:d:a	ns	ns	*	ns	ns	ns	ns	*	ns	-			
c:u:a	ns	ns	***	ns	ns	***	ns	*	ns	ns	-		
y:e:a	ns	ns	**	ns	ns	ns	ns	*	ns	ns	ns	-	
y:f:a	ns	***	**	ns	***	***	ns	ns	ns	ns	ns	ns	-

* 0.05 > p > 0.05; ** 0.05 > p > 0.005; *** p < 0.005; ns: non significant.

Table S7. Mean and standard deviation for SDS Sedimentation test (SDSS, mm), Grain Protein Content (GPC, %) and Thousand Kernel Weight (TKW, g) for a selected set of landraces, and their HMW-Gs composition

Accession id	SDSS (mm)	*Glu-A1*	*Glu-B1*	*Glu-D1*	GPC (%)	TKW (g)
**BGE012591**	100,83 ± 9,09	*b*	*e*	*a*	14,81 ± 2,35	29,72 ± 1,10
**BGE008229**	95,00 ± 30,25	*b*	7*+9	*d*	15,47 ± 3,44	30,30 ± 8,34
**BGE018228**	95,00 ± 17,94	*a*	*f*	*a*	16,37 ± 2,47	28,98 ± 11,04
**BGE008221**	91,13 ± 18,89	*b*	*7*+9*	*d*	15,30 ± 3,04	35,46 ± 7,36
**BGE012111**	90,88 ± 12,75	*c*	*u*	*a*	15,68 ± 2,60	27,51 ± 4,72
**BGE008205**	88,00 ± 24,42	*a*	*u*	*a*	14,64 ± 3,18	27,05 ± 4,29
**BGE012205**	84,88 ± 9,20	*c*	*e*	*c*	15,49 ± 2,72	31,28 ± 4,72
**BGE012292**	84,50 ± 29,44	*b*	7*+9	*d*	15,25 ± 2,79	28,83 ± 3,66
**BGE025410**	84,25 ± 21,01	*b*	*i*	*d*	13,76 ± 3,14	27,07 ± 7,12
**BGE025413**	81,88 ± 20,18	*b*	*u*	*a*	13,48 ± 2,48	29,63 ± 5,64
**BGE001944**	80,13 ± 15,79	*a*	*f*	*c*	14,81 ± 3,19	31,29 ± 9,78
**BGE012121**	77,13 ± 23,26	*b*	*u*	*a*	15,48 ± 3,14	32,42 ± 5,73
**BGE014290**	76,00 ± 32,36	*c*	*al*	*a*	16,59 ± 9,21	21,10 ± 5,17
**BGE024866**	75,75 ± 13,60	*a*	*f*	*c*	14,62 ± 2,35	30,94 ± 8,97
**BGE013795**	75,13 ± 18,09	*b*	*e*	*a*	13,61 ± 2,85	35,13 ± 9,64

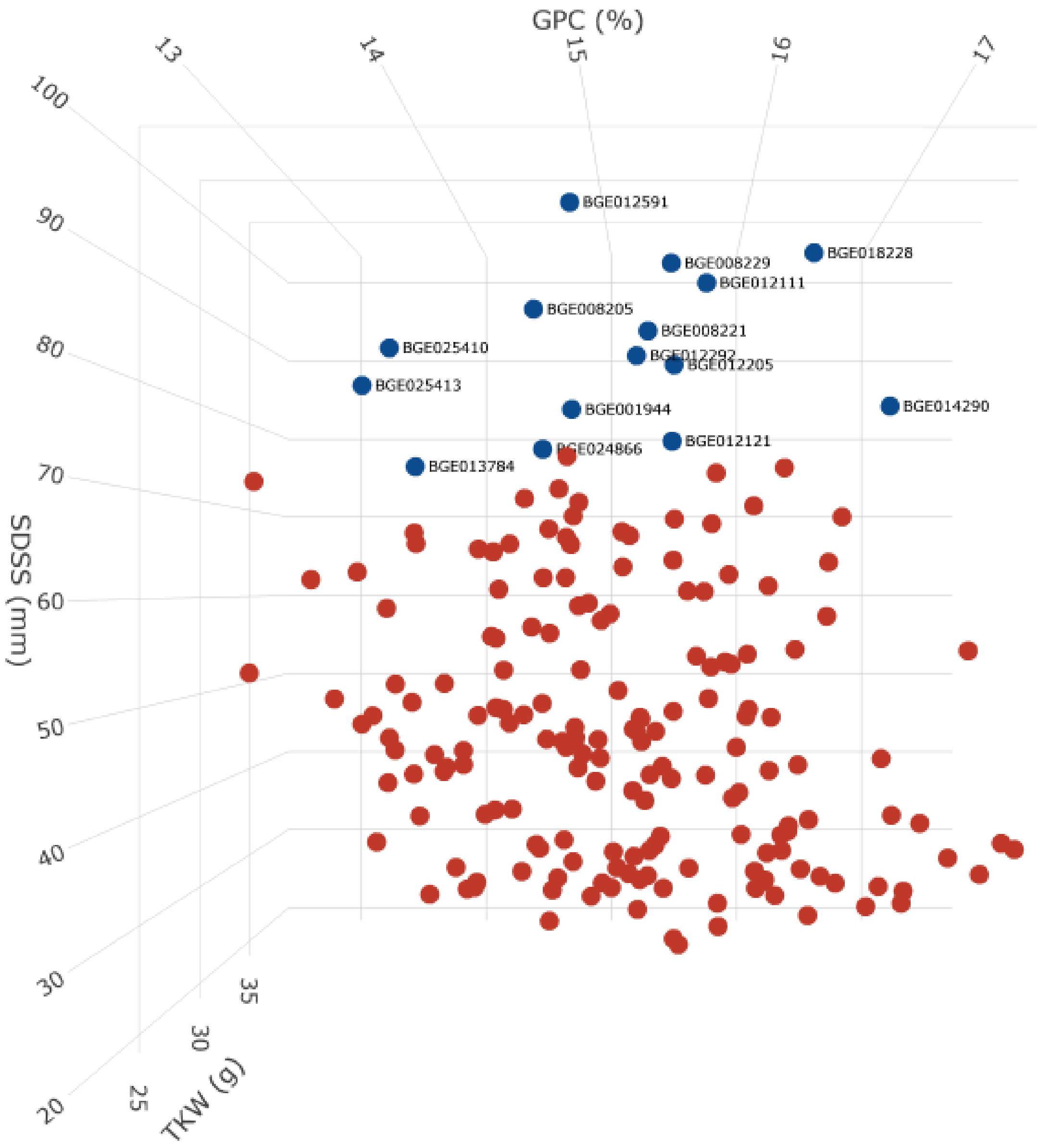

Figure S3. Set of fifteen landraces selected (in blue) based on SDS Sedimentation test values (SDSS, mm), Thousand Kernel Weight (TKW, g) and Grain Protein Content (GPC, %) to be further and deeply characterized in field trials.
